# Aquaporin 4 antibody‐positive neuromyelitis optica spectrum disorder with cholangiocarcinoma: Casual or causal association?

**DOI:** 10.1111/cns.14042

**Published:** 2022-12-16

**Authors:** Wei Fang, Jin‐Long Tang, Bin Lin, Qi‐Lun Lai, Meng‐Ting Cai, Zhi‐Rong Liu, Yin‐Xi Zhang

**Affiliations:** ^1^ Department of Neurology Hangzhou TCM Hospital Affiliated to Zhejiang Chinese Medical University Hangzhou China; ^2^ Department of Pathology, Second Affiliated Hospital, School of Medicine Zhejiang University Hangzhou China; ^3^ Department of Radiology, Second Affiliated Hospital, School of Medicine Zhejiang University Hangzhou China; ^4^ Department of Neurology Zhejiang Hospital Hangzhou China; ^5^ Department of Neurology, Second Affiliated Hospital, School of Medicine Zhejiang University Hangzhou China

Dear Editors,

Neuromyelitis optica spectrum disorder (NMOSD), preferentially affecting the optic nerves and spinal cord, is a representative inflammatory demyelinating disease of the central nervous system (CNS). The presence of antibodies targeting the water channel aquaporin 4 (AQP4) can be demonstrated in the serum of most patients.^1^ The exact etiology and pathogenesis of NMOSD have not been completely identified, and it is most commonly considered an idiopathic autoimmune condition, only a few cases have revealed that it can coexist with underlying malignancies and even emerge as a phenotype of paraneoplastic neurological syndrome (PNS).^2^, ^3^ Herein, we describe a 46‐year‐old woman who developed longitudinal extensive transverse myelitis (LETM) seropositivity for AQP4 antibody and a pathologically confirmed cholangiocarcinoma, which has not been reported before.

A 46‐year‐old woman complained of acute progressive bilateral lower extremity numbness and weakness, along with dysuria and constipation. Neurological examination revealed paraplegia (Medical Research Council [MRC] grade 0 in both legs) and remarkable sensory dysfunction below the nipple plane. A positive Babinski sign was observed on the left side.

Extensive laboratory workup was unrevealing, including tumor markers, pathogenic, inflammatory, and immunological assessments. Spinal magnetic resonance imaging (MRI) showed longitudinally extensive T2 hyperintense lesions involving the cervical and thoracic cord without enhancement (Figure 1). Chest computerized tomography and brain MRI were unremarkable. At the same time, abdominal ultrasound detected a mass in the left lateral segment of the liver. Cholangiocarcinoma was highly suspected according to the contrast‐enhanced MRI (Figure 1). Cerebrospinal fluid analysis revealed mild pleocytosis (8/μl) and a slightly elevated protein level (49.4 mg/dl). The oligoclonal band was negative. Further investigation demonstrated positive antibodies against AQP4 in serum (titer, 1:10) using a cell‐based assay.

**FIGURE 1:**
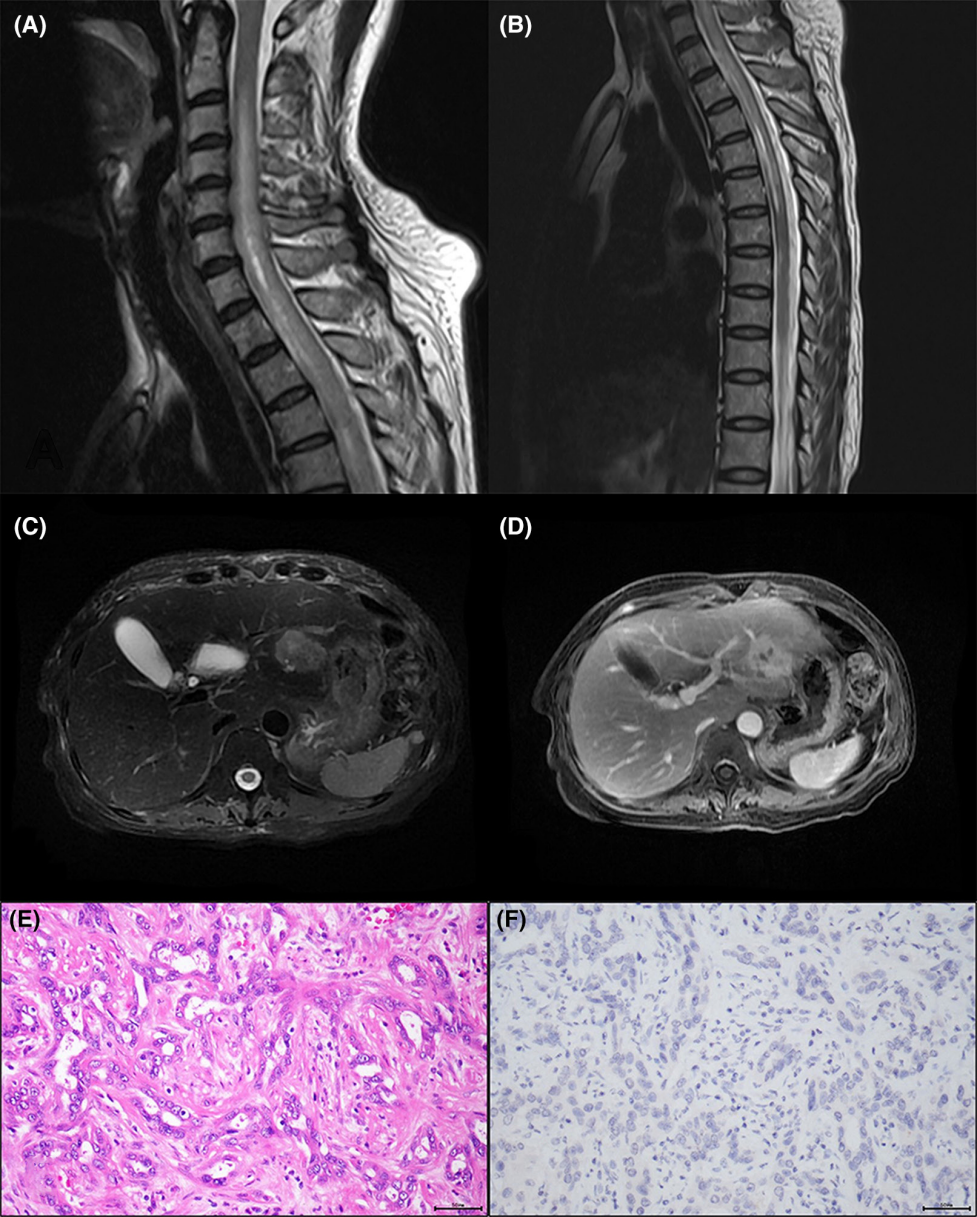
Sagittal magnetic resonance imaging (MRI) of cervical (A) and thoracic (B) spinal cord revealing longitudinal extensive T2 hyperintense lesions from C2 to T6. Hepatic MRI showing a focal T2 hyperintense nodular lesion (C) with contrast enhancement in the delayed phase (D) located in the left lateral segment of the liver. Hematoxylin–eosin staining demonstrating varying‐sized confluent, irregular‐angled gland composed of obvious atypia and vesicular nucleus, and pink cytoplasm, surrounded by abundant desmoplastic stromal reaction, suggesting poorly differentiated cholangiocarcinoma (E). Aquaporin 4 immunostaining was negative (F). Scale bars = 50 μm, magnifications 200×

The diagnosis of NMOSD was made. The patient was treated with intravenous methylprednisolone (1000 mg/day for 3 days, followed by a tapering scheme) and plasma exchange (3 cycles, every other day). Long‐term immunosuppressive therapy was suggested, but the patient refused.

The patient was then referred for surgery. The hepatic mass was removed, and postoperative pathological examination indicated a poorly differentiated intrahepatic cholangiocarcinoma. Immunohistochemical staining of the neoplastic tissue samples was performed, demonstrating the absence of AQP4 expression on the surface of tumor cells (Figure 1).

Unfortunately, no substantial improvement was observed after immunotherapy and oncotherapy (MRC grade 0 in both legs). Eighteen months later, at the latest visit, partial recovery of neurological disability was achieved (MRC grade 2 in both legs), and no neurological relapse and tumor recurrence were reported. The patient declined to repeat the AQP4 antibody test and spinal MRI during the follow‐up.

The LETM with seropositive AQP4 antibodies in this patient made the definite diagnosis of NMOSD based on the 2015 diagnostic criteria.^1^ Interestingly, asymptomatic cancer was detected through systemic screening. It was widely accepted that NMOSD often coexisted with systemic autoimmune diseases (e.g., Sjögren syndrome) with a strong association between them.^1^ However, accompanied by tumors in patients with NMOSD occur occasionally. In previous studies, the frequency of underlying neoplasms in NMOSD ranged from 3.2% to 7.1%, and the most commonly reported types were breast, lung, and genitourinary (mostly ovary) tumors.^4^ To the best of our knowledge, this is the first case report of AQP4 antibody‐positive NMOSD with concomitant cholangiocarcinoma.

The correlation between NMOSD and concurrent tumors remains uncertain. AQP4 is highly expressed in some tumors and may have a role in its migration. AQP4 antibodies are produced by the immune system to prevent tumors from spreading.^5^ However, in some cases, AQP4 antibodies may attack CNS by mistake, and patients will manifest as NMOSD. Positive AQP4 staining of tumor cells may mean that the tumors are the major cause of NMOSD in these patients.^6^ In other words, AQP4 antibody may be a type of paraneoplastic autoantibody, while NMOSD can be considered one of the phenotypes of PNS. On the other hand, negative AQP4 staining of tumor cells may mean that neoplasms are only coincidental findings independent of NMOSD, as in our patient. Moreover, a limited number of PNS with cholangiocarcinoma cases also support this incidental finding.^7^

According to the PNS diagnostic criteria defined in 2004, cancer present within 2 years of diagnosis established the possible paraneoplastic NMOSD in our patient.^8^ However, based on the 2021 updated criteria, our case is recognized as non‐PNS.^9^ This change is attributed to the new standard, which defines AQP4 antibody as low risk in association with cancer and would not identify as definite PNS even if tumor cells express AQP4 antigen recognized by the antibody.^9^ Regarding probable/possible paraneoplastic NMOSD, the diagnosis should be made with more caution and antigen expression demonstrated on the surface of tumor cells is necessary.

Treatment of NMOSD associated with tumors mainly comprises oncotherapy and immunotherapy. However, it remains controversial whether long‐term immunosuppression is required for these patients. Immunosuppressive agents have been prescribed to prevent future recurrence, including azathioprine, mycophenolate mofetil, rituximab, or tocilizumab.^10^ Whereas, improvement of neurological symptoms after acute‐phase corticosteroids and oncotherapy without further immunotherapy has been described.^3^ Additionally, a recent systematic review discovered that tumor resection had a significant relationship with complete recovery at the first attack and fewer relapses during follow‐up in patients with concomitant cancers.^4^ If the tumor and NMOSD attack has a cause‐and‐effect relationship, we speculate that maintenance treatment may not necessarily be warranted after effective oncotherapy. Future studies with large samples and long‐term follow‐ups are essential for clarification. In this case, inadequate improvement was noted despite prompt tumorectomy with stable cancer condition during follow‐up, which also supported that cholangiocarcinoma was an accidental phenomenon.

In conclusion, our patient with cholangiocarcinoma broadens the phenotypic spectrum of NMOSD associated with tumors. Although rare, a systemic screening for neoplasms is required in AQP4 antibody‐positive NMOSD patients with the first attack. If found, AQP4 staining of tumor cells is recommended to evaluate the potential role of the tumor in the development of NMOSD and determine the optimal therapeutic schedule for these patients.

## CONFLICT OF INTEREST

The authors declare that they have no conflict of interest.

## INFORMED CONSENT

Informed consent was obtained from the patient for publication of this paper.

## Data Availability

Anonymized data not published within this article will be made available upon reasonable request from any qualified investigator within 5 years after publication.
